# Rapid and Non-Destructive Techniques for the Discrimination of Ripening Stages in Candonga Strawberries

**DOI:** 10.3390/foods11111534

**Published:** 2022-05-24

**Authors:** Michela Palumbo, Rosaria Cozzolino, Carmine Laurino, Livia Malorni, Gianluca Picariello, Francesco Siano, Matteo Stocchero, Maria Cefola, Antonia Corvino, Roberto Romaniello, Bernardo Pace

**Affiliations:** 1 Institute of Sciences of Food Production, National Research Council (CNR), c/o CS-DAT, Via Michele Protano, 71121 Foggia, Italy; michela.palumbo@ispa.cnr.it (M.P.); antonia.corvino@ispa.cnr.it (A.C.); bernardo.pace@ispa.cnr.it (B.P.); 2 Department of Agriculture, Food, Natural Resources, and Engineering (DAFNE), University of Foggia, Via Napoli 25, 71121 Foggia, Italy; roberto.romaniello@unifg.it; 3 Institute of Food Science, National Research Council (CNR), Via Roma 64, 83100 Avellino, Italy; carmine.laurino@isa.cnr.it (C.L.); livia.malorni@isa.cnr.it (L.M.); gianluca.picariello@isa.cnr.it (G.P.); francesco.siano@isa.cnr.it (F.S.); 4 Department of Women’s and Children’s Health, University of Padova, 35131 Padova, Italy; matteo.stocchero@unipd.it

**Keywords:** *Fragaria* × *ananassa* Duch., e-nose, ATR-FTIR, image analysis, multivariate analysis

## Abstract

Electronic nose (e-nose), attenuated total reflection-Fourier transform infrared (ATR-FTIR) spectroscopy and image analysis (IA) were used to discriminate the ripening stage (half-red or red) of strawberries (cv Sabrosa, commercially named Candonga), harvested at three different times (H1, H2 and H3). Principal component analysis (PCA) performed on the e-nose, ATR-FTIR and IA data allowed us to clearly discriminate samples based on the ripening stage, as in the score space they clustered in distinct regions of the plot. Moreover, a correlation analysis between the e-nose sensor and 57 volatile organic compounds (VOCs), which were overall detected in all the investigated fruit samples by headspace solid-phase microextraction coupled to gas chromatography-mass spectrometry (HS-SPME/GC-MS), allowed us to distinguish half-red and red strawberries, as the e-nose sensors gave distinct responses to samples with different flavours. Three suitable broad bands were individuated by PCA in the ATR-FTIR spectra to discriminate half-red and red samples: the band centred at 3295 cm^−1^ is generated by compounds that decline, whereas those at 1717 cm^−1^ and at 1026 cm^−1^ stem from compounds that accumulate during ripening. Among the chemical parameters (titratable acidity, total phenols, antioxidant activity and total soluble solid) assayed in this study, only titratable acidity was somehow correlated to ATR-FTIR and IA patterns. Thus, ATR-FTIR spectroscopy and IA might be exploited to rapidly assess titratable acidity, which is an objective indicator of the ripening stage.

## 1. Introduction

Strawberries (*Fragaria* × *ananassa* Duch.) are worldwide popular fruits, highly appreciated for their colour, delicious taste, pleasant flavour and for their richness in nutrients, including flavonoids, anthocyanins, vitamin C and ellagic acid [1,2,3,4,5].

Usually, strawberries can be harvested at several ripening stages defined by colour changes, as red colour break, half-red and full red, depending on the final uses of the fruit [6]. However, since strawberries are non-climacteric fruits, it is necessary to harvest them at the optimum ripening stage to achieve the maximum quality in terms of taste, colour, consistency, size and shape.

Strawberry odour, which is strictly related to the consumer’s preference and to the sensory quality of the fruit, is the result of a complex mixture of multiple volatile organic compounds (VOCs) [4,5,7]. The profile of VOCs rapidly changes during ripening, but it is also affected by several other factors, including cultivar and pre- and postharvest handlings [7,8].

Currently, the harvest time is principally evaluated by counting the days after flowering, as well as by subjective tasting or visual evaluation of fruit colour, texture or plant canopy structure. All these methods on their own or in combination lack accuracy and objective evaluation [9]. Chromatographic techniques (i.e., gas chromatography) coupled with different detectors (e.g., mass spectrometer) are suitable analytical techniques to estimate fruit ripeness and quality on a molecular basis. Although these tools present several advantages in terms of reliability, selectivity and ability to detect the classes of compounds contained in the sample, in general they are destructive, time consuming, expensive, require skilled personnel, polluting and unable to analyse the entire batch of products, since only a small more or less representative sample of fruits can be inspected [6].

In recent years, time-/cost-effective, non-destructive, contactless, user-friendly and green non-targeted methods have been increasingly introduced to perform fingerprinting studies aimed at monitoring the degree of ripening and the quality traits of fruit on an objective basis. The practical advantages of these methods consist of minimizing potential losses for growers and packers, as well as fast decay for the end consumer. The quality parameters or the index of ripening assessed by non-destructive methods can also be designed to predict the optimal harvest time [9].

Fourier transform infrared (FTIR) spectroscopy [10,11,12], image analysis (IA) [13,14,15] and electronic nose (e-nose) [16,17] are among the main approaches that have been developed to assess real time the state of ripeness and the internal and external quality appearance of fruit.

Specifically, IA technique has proved to be a successful contactless tool in the quality assessment of fruits and vegetables. This technology acquires images of the whole visible surface of the products, extracts the most discriminative external features (shape, colour and defects) and processes the data by regression or classification models and algorithms to predict chemical and physical properties of the samples [18].

For example, IA and machine learning techniques have been used to recognize mature strawberries from shape and colour characteristics extracted from the images acquired [14]. Moreover, ref. [19] proposed an innovative low-cost computer vision based on convolutional neural networks for strawberry detection to implement and develop a contactless and non-destructive robot for fruit harvesting. 

Fruity aroma is another key indicator of strawberry quality, which is strictly related to the fruit ripeness. VOCs and phenolic compounds have been recently profiled in “Candonga” strawberries, comparing fruits harvested at half-red and red ripening stages [20]. Some VOCs, namely butyl butyrate, ethyl hexanoate, hexyl acetate, nonanal, terpenes and lactones, were individuated as putative markers of the maturity phase at harvest.

E-nose represents one of the most promising, fast, easy-to-handle, low cost and non-destructive methods, as an alternative to conventional ones (i.e., headspace solid-phase microextraction (HS SPME) sampling followed by gas chromatography-mass spectrometry (GC-MS)) for the detection of food odour, aimed at discriminating and classifying food matrices with different aroma fingerprints. E-nose devices comprise an array of chemical sensors, which offer selectivity toward varying classes of VOCs, collecting aroma information of a sample as a whole. This technique can simulate the human olfactory sense and generate fingerprints of VOCs in real time [21]. E-nose sensory data combined with suitable chemometric tools have been successfully applied to detect changes in VOC content in several food matrices for several purposes, including food quality, safety and fraud detection as well as to discriminate the fruit based on its degree of ripening [7,21,22,23]. 

E-nose equipped with metal oxide gas sensors was successfully employed to characterize the volatile patterns of Festival and Florida Radiance strawberry cultivars at five ripening stages: white, half red, three-quarter red, full ripe and overripe [16]. Results of this study could provide data reproducibility of 90% (±10%), demonstrating the potentiality of this technology to monitor strawberry maturity and fruit quality. Ref. [17] reported the use of a new self-developed e-nose system to detect strawberry freshness during postharvest. This system consisted of different metal oxide semiconductor sensors linked to a data acquisition system and to a computer with pattern recognition software. The e-nose response values detected during the storage were used to build both a partial least squares (PLS) discriminant analysis and a support vector machine (SVM) model, demonstrating that SVM was a better model than the PLS one, providing an accuracy of 96.3% and 94.9% for the training and testing sets, respectively.

FTIR coupled with chemometrics has been extensively explored as a powerful, rapid and time-/cost-effective analytical technique for studying the chemical composition of fruit, also offering specific information about their dynamic changes. More recently, attenuated total reflection (ATR)-FTIR techniques have been exploited to simultaneously determine attributes of quality, bioactive compounds and antioxidant capacity of ten strawberries cultivars harvested at seven different stages of ripening [12]. FTIR has also been successfully exploited to monitor strawberry spoilage [10].

In this research paper, the applicability of rapid and non-destructive techniques, including e-nose, ATR-FTIR and IA, combined with chemometric methods, for the fast and reliable discrimination of two ripening stages (half-red and red) in “Candonga” strawberries, the most cultivated in Europe, was investigated. The final aim was to establish the most suitable rapid methods for assessing strawberry ripening, through correlation with one or more objective quality indicators of ripening (VOCs, titratable acidity (TA), total phenols (TP), antioxidant activity (AA) and total soluble solid (TSS)). 

## 2. Materials and Methods

### 2.1. Plant Material

Candonga strawberries (*Fragaria* × *ananassa* Duch.) var. Sabrosa (Planitalia s.r.l., Policoro, Italy), packed into PET trays (Carton Pack SpA, Rutigliano, Italy), were provided by a cooperative company of fresh fruit (Apofruit Italia Soc. Coop., Scanzano Jonico, Italy) at three different consecutive harvest times, on 21 May, 27 May and 1 June, indicated as H1, H2 and H3, respectively, and at two maturity stages, thereinafter indicated as half-red (in ripening phase, fully expanded and 50% red) and red (in ripening phase, fully expanded and 100% red), consistent with the visual criteria reported by [20] (Figure S1). Total soluble solids and titratable acidity at harvest were about 8.8 ± 0.7 °Brix and 0.9% for half-red strawberries and 9.8 ± 0.2 °Brix and 0.7% for red ones. The same samples have been previously subjected to the analysis of VOCs and individual phenolic compounds and to the determination of several chemical parameters, including titratable acidity (TA), total phenols (TP), antioxidant activity (AA) and total soluble solid (TSS) as reported in [20]. In this work, these samples have been analysed by e-nose, ATR-FTIR and IA. 

### 2.2. Electronic Nose (E-Nose)

Comprehensive profiles of VOCs from the headspace of “Candonga” strawberry samples were obtained using a commercial portable electronic nose (e-nose, PEN 3, Airsense Analytics Inc., Schwerin, Germany, including the Win Muster software). The sensor array was fitted out with 10 metal oxide semiconductor (MOS) sensors characterized by different thicknesses and chemical compositions, to offer selectivity toward different classes of volatile, as described by [24]. Owing to the high operative temperatures (200–500 °C), VOCs delivered to the surface of the sensors were completely converted to carbon dioxide and water, causing a variation in the resistance. The response of the MOS sensors, revealed as resistivity (O), was established on the changes in conductivity, due to the adsorption of gas molecules, and on the subsequent surface reactions. For sample preparation, 4 g of each sample were placed in 45 mL airtight glass vials and closed with a screw cap with poly (1,1,2,2-tetrafluoroethylene) (PTFE)/silicone septum. To get the headspace equilibrium, each vial was held for 30 min at 30 °C and detections were performed at a temperature of 22 ± 2 °C and a relative humidity (RH) of 50 ± 5%. To minimize the drift in MOS sensors, each sampling involved a measurement process of 80 s to get the stable signals followed by a 70 s cleaning process to normalize the sensor responses, in line with preliminary experiments. The sample gas was driven into the sensor chamber at a flow rate of 400 mL/min. For each sample, analyses were carried out in 11 technical replicates and the signals per second were collected. The signals of each sensor were expressed by the ratio G/G0, where G and G0 indicate the conductance of the sensors exposed to sample gas and to the clean gas, respectively. Successively, to clean the system between two sequential analyses, a second pump transports the filtered air to the sensor array for 400 s with a flow rate of 600 mL/min. E-nose data were registered by the pattern recognition software (WinMuster, v.1.6., Airsense Analytics GmbH, Schwerin, Germany) and the average response of each sensor in the range 70–75 s (area under the curve) was submitted to statistical data analysis.

### 2.3. Attenuated Total Reflection-Fourier Transform Infrared (ATR-FTIR) Spectroscopy 

Sets of at least 10 strawberries of each maturity stage were randomly sampled, homogenized with a blender, frozen in dry ice and finally lyophilized. The resulting solids were finely powdered using a ceramic mortar. ATR-FTIR spectra of powders were acquired using a Spectrum 400 spectrophotometer (PerkinElmer, Waltham, MA, USA), with a Deuterated Triglycine Sulfate (DTGS) detector. The sampling station was equipped with an overhead ATR accessory. The spectra were recorded in the 650–4000 cm^−1^ region at 32 scans/spectrum range and with resolution of 4 cm^−1^. Analyses were performed in triplicate and averaged spectra were processed using the PE Spectrum software (PerkinElmer, Milan, Italy, version 10.5.1).

### 2.4. Image Analysis (IA)

At each harvest time and for each maturity stage (half-red or red), a lot containing 12 strawberries for each replicate was imaged using a Digital Camera AP-3200T-PGE (JAI Ltd., Yokohama, Japan) positioned inside a Photo studio box HPB-60D (HAVOX^®^, Vendôme, France). In total, for each maturity stage, four replicates were considered, for a total of 48 berries. The camera sensor was composed of three CMOS RGB-sensors, delivering a spatial resolution of 3.2 MPixels @12 frame/second and a colour depth of 24 bit/pixel. The objective used was a 12 mm focal and F1.8 (KOWA Lens mod. LM12NC3 1/2) allowing a field of view (FOV) of (35 × 30 cm). Illumination was provided by two led ramps with 120 lamps (HAVOX HPB-60, 5500K, 13,000 ± 100 lumen CRI 93+). A ColourChecker Passport Photo 2 (X-rite Italy srl, Prato Italy) with 24 patches of known colour was placed in the camera FOV as the chromatic reference.

Images were pre-processed and analysed using the Image Processing Toolbox of Matlab software R2021b (MathWorks Inc., Natick, MA, USA). The algorithm enabled us to acquire the raw images and pre-process them by cropping the unnecessary image border and separating the three colour-components, red, green and blue (RGB), from the original raw images. The background was thresholded using the R image, since having the highest contrast with the black background. The coarse segmentation of the strawberries was carried out by a threshold method [25]. On the obtained binary images, a morphological filter was applied to erode the strawberry edge and a flood filling operation was performed to overcome the thresholding defects. Using this primary mask, a secondary segmentation was performed to individuate the green and red areas. To this topic an enhanced image was obtained by subtracting the G image to R image. The R image was thresholded and a secondary mask was gained, obtaining the red area. The green area was simply obtained by subtracting the secondary to the primary mask. Finally, the pixel count of each area was performed to calculate the red and green percentage area.

### 2.5. Total Soluble Solids, Titratable Acidity, Antioxidant Activity and Total Phenols

Total soluble solids (TSS) and titratable acidity (TA) were measured on about 100 g of homogenized strawberries (for each replicate and maturity stage) as reported by [20]. 

For each replicate and at each ripening stage, about 100 g of strawberries were homogenized to obtain the fruit juice on which the measurements of pH have been performed. The TSS content was determined using a digital refractometer (DBR35-XS Instruments, Carpi, Italy) and results were expressed in °Brix. 

The analysis of antioxidant activity (AA) and the total phenols (TP) was performed with the spectrophotometric method previously described by [20] using a spectrophotometer UV-1800, (Shimadzu, Kyoto, Japan).

In detail, antioxidants were extracted in 20 mL methanol/water solution (80:20 *v*/*v*) for 2 min, using a homogenizer (T-25 digital ULTRA-TURRAX^®^—IKA, Staufen, Germany). Then, AA was measured on the methanol extract using the DPPH (1,1-diphenyl-2-picrylhydrazyl) assay, while TP were determined by Folin–Ciocalteu’s method. 

### 2.6. Analysis of Volatile Compounds (VOCs)

Identification and semi-quantification of VOCs was carried out by HS-SPME/GC-MS as previously reported by [20] using a DVB/CAR/PDMS (50/30 μm) fibre and 50 °C and 20 min as extraction temperature and time, respectively. VOC analysis was performed by using the GC system, model GC 7890A, Agilent (Agilent Technologies, CA, USA), coupled to the mass spectrometer 5975C (Agilent). Semi-quantitative data of each volatile (relative peak area, RPA%) were measured in respect to the peak area of 2-octanol, used as the internal standard [20]. Peak areas of the identified VOCs were calculated from the total ion chromatogram (TIC).

### 2.7. Statistical Data Analysis

Data were investigated by univariate and multivariate data analysis. In detail, in univariate data analysis a linear mixed effect model (LME), in which the maturity stage was assumed as the fixed factor and the technical replicate as the random factor, was considered, in order to evaluate the effect of the maturity stage. False discovery rate (FDR) was controlled by the Benjamini–Hochberg procedure assuming a level δ = 0.05. Moreover, the receiver operating characteristic (ROC) curve was used as a tool to estimate the capability to distinguish the maturity stage based on the data. Multivariate data analysis was based on principal component analysis (PCA) [26].

The correlations among e-nose responses and VOCs and between chemical data and IA and ATR-FTIR data were explored by correlation analysis. In particular, the correlation matrices based on the Pearson correlation coefficient were explored by heatmap, using a hierarchical clustering procedure that used Euclidean distance and Ward’s method. A significance level α = 0.05 was assumed for the correlation coefficients. The optimal number of clusters was determined by silhouette analysis. Data analysis was carried out by in house R-function executed using the R 4.0.4 platform (R Foundation for Statistical Computing, Vienna, Austria).

## 3. Results and Discussion

### 3.1. E-Nose Discrimination of the Ripening Stage of “Candonga” Strawberries and Correlation Analysis with VOC Pattern 

Four lots of 12 “Candonga” strawberries were obtained pooling the fruit collected for each harvest time (H1, H2 and H3) at the two different ripening stages (half-red and red), obtaining a total of 12 samples for the half-red and 12 samples for the red fruit. Since 11 technical replicates were performed for each sample, a data set composed of 264 observations and 10 variables (sensor responses) was investigated by univariate data analysis and the results are reported in Table 1. All sensors, except for S4 and S10, resulted in us being able to distinguish the two maturity stages.

**Figure 1:**
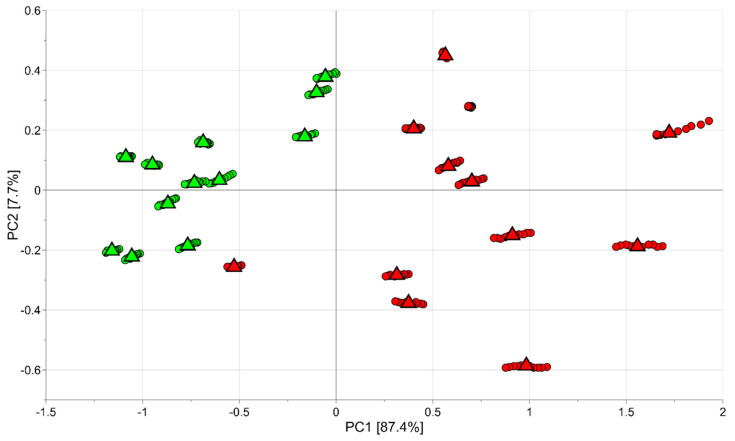
PCA model of the e-nose data: circles represent the observations (technical replicates), while triangles indicate the medians of the technical replicates of the same sample; the green colour is used for observations belonging to the half-red group and the red colour for those of the red group.

The same data set (264 observations and 10 variables) was mean centred and investigated by PCA. A model with two principal components with R^2^ = 0.95 and Q^2^ = 0.81 was obtained. In particular, in the score scatter plot of the model shown in Figure 1, where half-red samples are indicated in green and red samples are reported in red, the data variation due to technical replicates (indicated by the circles) was smaller than the biological variability (the medians of the technical replicates of the same sample are indicated by the triangles). Moreover, the samples at different ripening stages were easily classified into two groups based on their degree of ripeness (red and half-red). Interestingly, half-red samples were spread over a smaller area than the region of the plot relevant to the red strawberries. This result indicates a larger variability among the red than the half-red fruit.

The PCA results indicated that there was a potential relationship among the e-nose signals and the ripeness of fruit, as the e-nose device responded sensitively and selectively to the modification in the patterns of aroma VOCs from strawberries at different ripening stages.

Overall, 57 volatile compounds were previously identified by HS-SPME/GC-MS analysis of the “Candonga” strawberry samples at two different ripening stages (half-red and red) and at three different harvest times (H1, H2 and H3) (Table S1) [20]. As reported by [20], PCA performed on volatiles, quality traits and phenolic com-pounds highlighted that only the red samples were directly correlated to volatile components, as VOCs clearly increased both in number and amount during ripening. In particular, volatiles with positive impact on the consumers’ acceptance, including butyl butyrate, ethyl hexanoate, hexyl acetate, nonanal, terpenes and lactones, were positively associated to the red-H1 and red-H2 strawberries, while volatiles with negative coefficients related to consumer liking, including isopropyl butyrate, isoamyl butyrate and mesifurane, were directly correlated to the red-H3 samples

**Figure 2:**
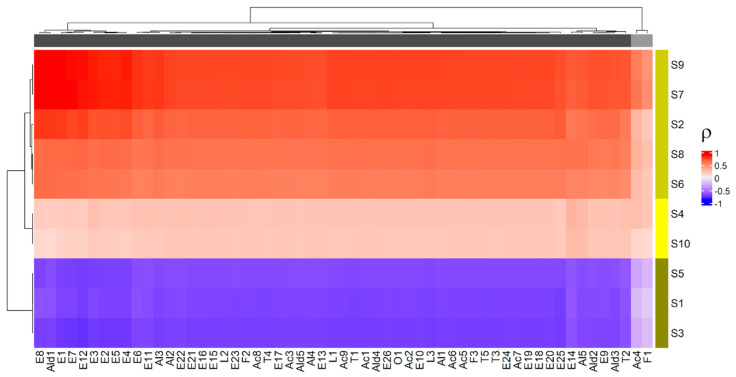
Heatmap: a different colour code was used to represent the clusters discovered by cluster analysis; ρ is the Pearson correlation coefficient.

All these VOCs were correlated with the data obtained by e-nose on the same fruit samples. The heatmap representing the correlation between VOCs and e-nose is reported in Figure 2. 

Cluster analysis allowed us to gather the VOCs in two groups and the e-nose sensors in three clusters. Specifically, the first group consisted of all the VOCs with the exception of 2-methylbutanoic acid (Ac4) and mesifurane (F1), which formed the second cluster (Figure 2). On the other hand, the first group of the e-nose sensors included S2, S6, S7, S8 and S9, the second involved S1, S3 and S5 and the third cluster contained S4 and S10. Specifically, the first group of VOCs exhibited a highly positive association with the sensors S2 (broad range), S6 (broad-methane), S7 (sensitive to many terpenes), S8 (broad-alcohol) and S9 (responsive to sulphur and aromatic metabolites) and a highly inverse correlation with the sensors S1 (sensitive to aromatic compounds), S3 (sensitive to ammonia and aromatic compounds) and S5 (sensitive to hydrocarbons and aromatic compounds). The correlations with the sensors S4 (sensitive to hydrogen) and S10 (sensitive to methane) were negligible, in agreement with the univariate data analysis (Table 1). Moreover, the sensors of the first cluster (S2, S6, S7, S8 and S9) presented a higher response to the red fruit, which may be caused by the increase in methane and alcohol, in line with previous studies [16]. On the other hand, the sensors of the second cluster (S1, S3 and S5) showed a higher signal to the half-red samples, allowing us to discriminate between the two ripening stages (Table 1, Figure 2). 

The compounds Ac4 and F1 were positively correlated to the sensors S7 and S9 (Figure 2), and their correlation coefficient showed smaller absolute values with respect to the other VOCs, maintaining the same sign. Interestingly, in our previous study these two compounds showed a significant correlation only with red-H3 strawberries [20]. Specifically, mesifurane (F1), which is known to increase during fruit ripening and to confer caramel or cotton candy-like notes to ripe strawberries, has been reported to have a negative impact on the consumers’ acceptance [27]. Overall, our results highlighted that e-nose responses were in line with HS-SPME/GC-MS analysis. In addition, e-nose sensors were able to discriminate “Candonga” strawberries at the two different ripening stages by differently reacting to specific VOCs from the different fruit samples.

### 3.2. ATR-FTIR Discrimination of the Ripening Stage of “Candonga” Strawberries and Correlation Analysis with Chemical Data 

**Figure 3:**
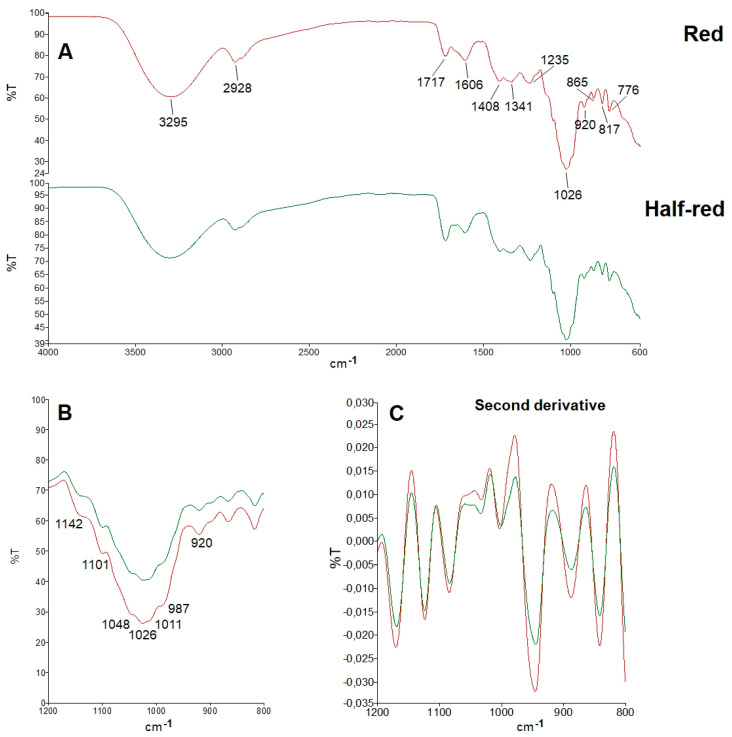
(Panel (**A**)): Typical ATR-FTIR spectrum in the 4000–600 cm^−1^ range of freeze-dried red (red line) and half-red (green line) strawberries. (Panel (**B**)): Magnified view of the spectral fingerprint region (1200–800 cm^−1^), dominated by vibrational modes of sugars. (Panel (**C**)): Second derivative of spectra in the 1200–800 cm^−1^.

Spectral sub-ranges identifying the main strawberry constituents could be detected within the exemplary ATR-FTIR full range spectra (4000–600 cm^−1^) of half-red and red samples (Figure 3A). The broad band centred at 3295 cm^−1^ and those at 2928 and 2891 cm^−1^ corresponded to the νOH and νCH vibrational modes, respectively. The band at 1717 cm^−1^, which is diagnostic of the νC=O vibrational mode of esters or organic acids, revealed the methyl galacturonates of strawberry pectins. The bands at around 1400 cm^−1^ arose from the δCH_2_ vibrational modes [11].

A magnified view of the spectral region in the fingerprint section (1200 and 800 cm^−1^) containing the specific bands of sugars is shown in Figure 3B. The main vibrational modes in this region include the δC–O–C of the glycosidic linkage, δCOH, and νC–C. Clearly, this region is characterized by overlapping bands resulting from the contribution of all the different carbohydrates [11]. The various intensity of the bands, which emerges with stronger evidence from the second derivative of spectra in the 1200–800 cm^−1^ range (Figure 3C), reflected the dynamic concentration changes of the various sugars during strawberry ripening.

**Figure 4:**
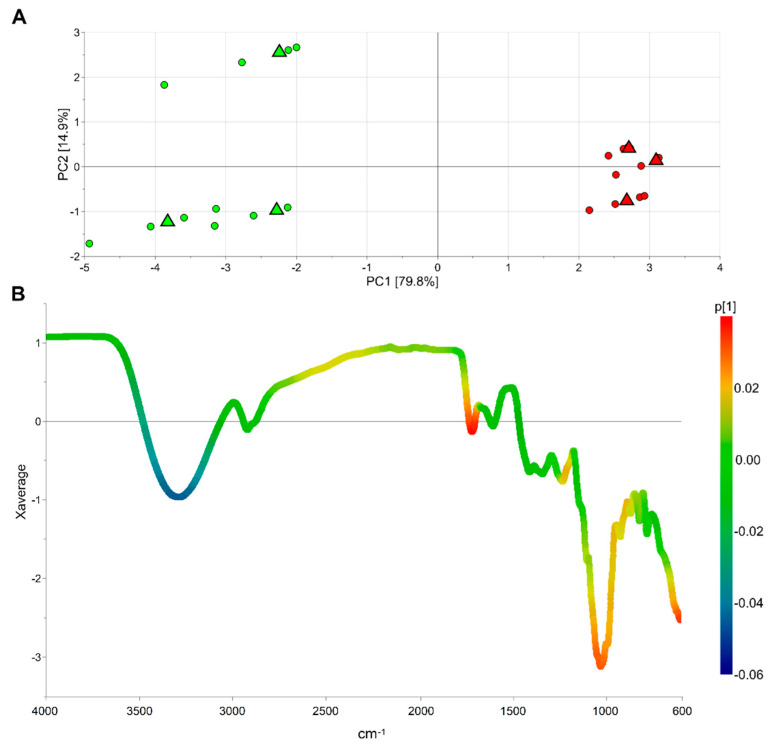
PCA model of the ATR-FTIR data: score scatter plot (panel (**A**)) and average profile of the SNV data coloured according to the first loading vector (panel (**B**)); in panel A, circles represent the technical replicates, triangles indicate the medians of the technical replicates of the same sample and green is used for observations belonging to the half-red group while red is used for those of the red group.

Six different samples of “Candonga” strawberries were obtained pooling the collected fruits for each harvest time and the two different ripening stages, gaining a total of three samples for the half-red and three samples for the red fruit. Five technical replicates were performed for each sample obtaining a data set composed of 30 observations and 3401 variables (wavenumber). The PCA model obtained after standard normal variate (SNV) transformation and by mean centring the data showed two principal components with R^2^ = 0.947 and Q^2^ = 0.941. The score scatter plot of the model is reported in Figure 4A. PCA1, explaining the 79.8% of the total variance, discriminates the samples belonging to the half-red group from those of the red group. As a result, the loading of the first component can be investigated to discover the IR spectral regions capable to distinguish the two groups. Specifically, Figure 4B allows us to observe that the average spectrum is coloured according to the loading value. Moreover, the broad band centred at 3295 cm^−1^, the band at 1717 cm^−1^ and the band at 1026 cm^−1^ were the most related to ripening. The compounds responsible for the signals of the first band decrease their concentration during fruit ripening, while the others increase their concentration along with strawberry maturation. The broad band centred at 3295 cm^−1^ corresponds to the νOH vibrational mode of organic acids, while the band 1717 cm^−1^ can be ascribed to the νC=O vibrational mode of esters or organic acids. Moreover, the 1200 to 900 cm^−1^ spectral range, which includes the band at 1026 cm^−1^, is known as the “fingerprint region” of sugars [11]. The main vibrational modes absorbing in this region correspond to the δC–O–C of the glycosidic linkage, the δCOH, and the νC–C of sugars and organic acids, both affected by the ripening stage of strawberries. Indeed, during fruit ripening the concentration of organic acids tends to drop, while that of sugars (sucrose, glucose and fructose) is likely to increase [28].

**Figure 5:**
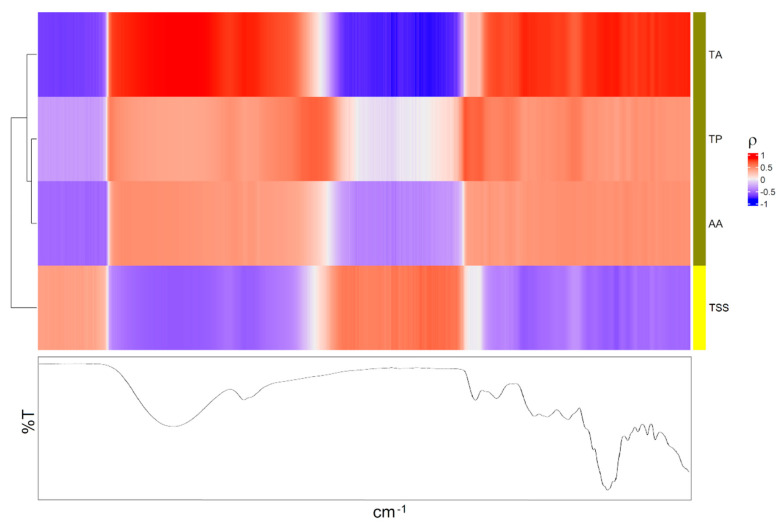
Correlations between ATR-FTIR and chemical data: heatmap; a different colour code was used to represent the clusters discovered by cluster analysis; ρ is the Pearson correlation coefficient; in the bottom, the average IR spectrum is reported.

The relationships between ATR-FTIR data and the chemical parameters including TA, TP, AA and TSS, detected as reported in [20] (Table S2), were assessed by the heatmap of Figure 5. Two clusters were discovered for the chemical data. Specifically, TA, TP and AA behave similarly being positively correlated to the IR signals in the regions 3650–2700 cm^−1^ and 1500–600 cm^−1^, while TSS showed an opposite trend. Only TA resulted to be significantly correlated to the ATR-FTIR data and, in particular, to the two bands in the region 3650–2700 cm^−1^ and with the peaks in the regions 1500–1280 cm^−1^ and 1160–700 cm^−1^. In other words, TA resulted to be the chemical attribute more related to IR data and the IR spectra mirrored the behaviour of TA. Generally, TA decreases during ripening due to the conversion of organic acids into sugars [29], and it is an objective indicator of maturity stage. Consequently, its prediction by ATR-FTIR might be a valid solution for a fast and non-destructive assessment of the ripening stage in strawberries.

### 3.3. Image Analysis Discrimination of the Ripening Stage of “Candonga” Strawberries and Correlation Analysis with Chemical Data

Four samples of 12 “Candonga” strawberries were obtained pooling the collected fruit at each harvest time and at two different ripening stages, achieving a total of 12 samples for the half-red and 12 samples for the red fruit. Eight technical replicates were performed for each sample obtaining a data set composed of 192 observations and five variables (measuring colour attributes). All colour parameters obtained by strawberry image analysis allowed us to significantly discriminate the half-red and the red samples, as reported in Table 2. 

**Figure 6:**
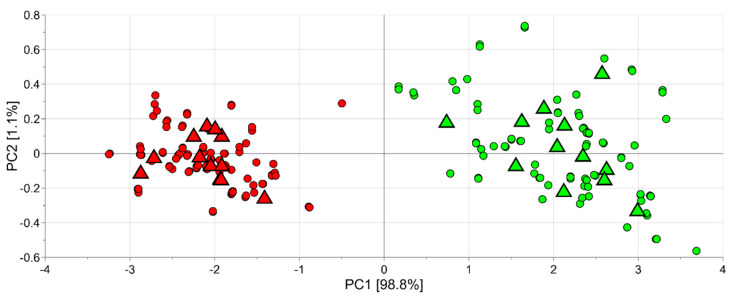
PCA model of the image data: circles represent the observations (technical replicates), while triangles indicate the medians of the technical replicates of the same sample; green is used for observations belonging to the half-red group and red for those of the red group.

Indeed, for all the colour parameters considered the half-red samples showed median values significantly higher than red ones at each harvest time (fold changes greater than 1). This result is clearly visible also looking at the score scatter plot obtained by PCA analysis (autoscaled data, two principal components, R^2^ = 0.999), using as variable the colour data (Figure 6). Red and half-red strawberries clustered in two different regions, at the right-hand side and at the left-hand side along the first component, respectively.

**Figure 7:**
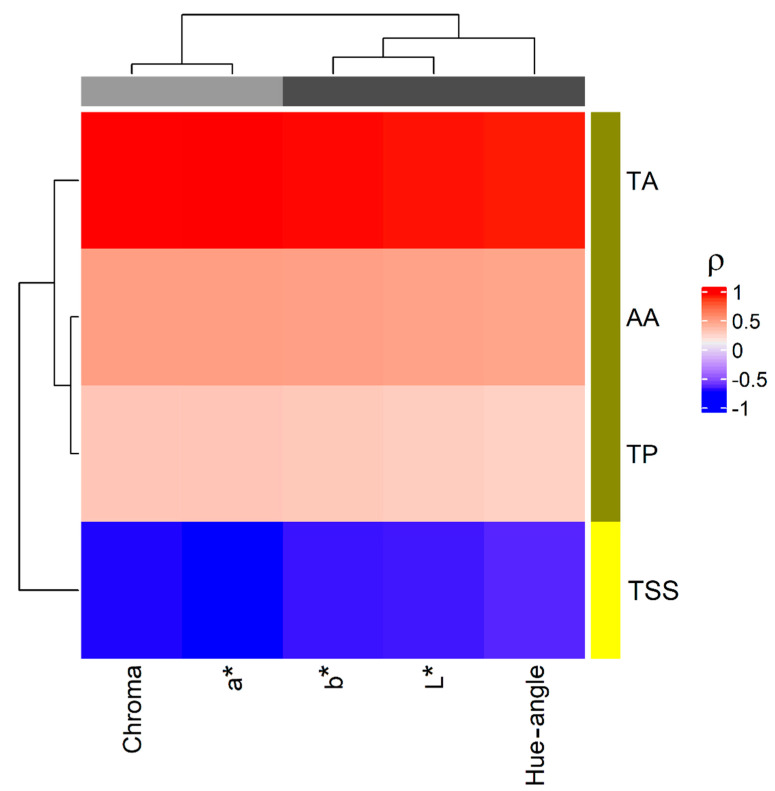
Correlations between image data and chemical data: heatmap; a different colour code was used to represent the clusters discovered by cluster analysis; ρ is the Pearson correlation coefficient.

The relationships between image data and the chemical attributes (TA, TP, AA and TSS) were explored by heatmap (Figure 7). As in the case of ATR-FTIR data, two clusters were discovered for the chemical data. Specifically, TA, TP and AA behave similarly being positively correlated to the colour parameters, while TSS showed an opposite trend, being negatively correlated to the colour parameters. On the other hand, the colour parameters were grouped into two clusters, one formed by Chroma and a* and the other including b*, L* and Hue-angle. Only TA resulted to be significantly correlated to the image data that mirrored its trend. The development of fruit colour during ripening is considered a maturity index and when its intensity increases, the ripening attributes also improve. As the fruit colour development enhances, the TSS content increases proportionately. On the contrary, the trend of the fruit acidity decreases with the enhancement of the colour of fruit during ripening [30]. This result suggests that the prediction of TA by the colour parameters obtained by IA of the strawberry represent a valid method to rapidly assess the ripening stage.

## 4. Conclusions

E-nose, ATR-FTIR and image analysis were used as rapid and non-destructive techniques to discriminate the ripening stage (half-red or red) of strawberries cv Sabrosa, commercially named Candonga, harvested at three different times. The correlation analysis between e-nose sensor responses and VOC data demonstrated that HS-SPME/MS-e-nose experimental data contain enough information to allow the discrimination of strawberry samples based on their degree of ripening. Moreover, among the chemical indicators of ripening, TA was correlated to both ATR-FTIR and IA data. Since TA usually decreases during ripening, its assessment by ATR-FTIR or IA might provide a suitable indicator for a fast and non-destructive evaluation of the ripening stage in strawberries.

Further investigations to improve the results obtained herein, performed using a larger sample size and cultivars as well as samples of different strawberry crop seasons, will be carried out with the aim to build predictive models of ripening, which can offer several advantages in terms of rapidity, low cost and easy-to-handle analysis for the industries of the sector. Models built considering multiple techniques (i.e., ATR-FTIR and IA) will also be considered in the perspective of improving the reliability of the models. The final goal is to obtain significant models usable in portable devices for a real-time and on-site prediction of the most suitable harvest time.

## Figures and Tables

**Table 1:** Univariate data analysis: ID indicates the sensor name, in columns half-red and red the median (5th–95th) percentile is reported for the two maturity stages, *p* is the *p*-value of the factor maturity stage obtained by LME, FC is the fold change calculated as the ratio between the median of red and the median of half-red, AUC is the area under the ROC curve and CI 95% is the confidence interval of AUC at the level of 95%.

ID	Half-Red	Red	*p*	FC	AUC	CI 95%
S1	0.439 [0.415–0.456]	0.408 [0.364–0.429]	<0.001	0.929	0.946	0.922–0.969
S2	3.812 [3.416–4.185]	4.710 [3.924–5.541]	<0.001	0.809	1.236	0.950–0.989
S3	0.430 [0.409–0.443]	0.389 [0.359–0.414]	<0.001	1.105	0.905	0.942–0.98
S4	1.075 [1.061–1.084]	1.077 [1.067–1.085]	0.40	0.998	1.002	0.506–0.646
S5	0.421 [0.398–0.443]	0.380 [0.356–0.406]	<0.001	1.108	0.903	0.940–0.98
S6	3.907 [3.691–4.225]	4.198 [3.845–4.669]	0.003	0.931	1.074	0.810–0.903
S7	1.322 [1.234–1.445]	1.930 [1.506–2.198]	<0.001	0.685	1.460	1.000–1.000
S8	5.713 [5.349–6.303]	6.370 [5.671–6.974]	<0.001	0.897	1.115	0.847–0.924
S9	1.800 [1.642–1.95]	2.283 [2.042–2.555]	<0.001	0.788	1.269	1.000–1.000
S10	1.196 [1.160–1.224]	1.204 [1.172–1.226]	0.40	0.994	1.006	0.526–0.669

**Table 2:** Univariate data analysis of image data: ID indicates the feature name, in columns half-red and red the median (5th–95th) percentile is reported for the two maturity stages, *p* is the *p*-value of the factor maturity stage obtained by LME, FC is the fold change calculated as the ratio between the median of red and the median of half-red, AUC is the area under the ROC curve and CI 95% is the confidence interval of AUC at the level of 95%.

ID	Half-Red	Red	*p*	FC	AUC	CI
L*	15.71 [13.61–16.81]	10.58 [9.78–11.40]	<0.001	0.676	1.00	1.00–1.00
a*	25.78 [23.28–27.46]	20.23 [19.02–21.58]	<0.001	0.787	1.00	1.00–1.00
b*	16.40 [14.03–17.72]	10.16 [9.14–11.31]	<0.001	0.621	1.00	1.00–1.00
Chroma	30.51 [27.23–32.56]	22.62 [21.20–24.36]	<0.001	0.741	1.00	1.00–1.00
Hue-angle	0.56 [0.53–0.59]	0.46 [0.44–0.48]	<0.001	0.826	1.00	1.00–1.00

## Data Availability

Not applicable.

## Supplementary Materials

The following supporting information can be downloaded at: https://www.mdpi.com/article/10.3390/foods11111534/s1, Table S1: Volatiles compounds obtained in strawberries cv Sabrosa, commercially named Candonga at two different ripening stages (half-red and red) and at three harvest times (H1, H2 and H3); Table S2: Chemical parameters measured in strawberries cv Sabrosa, commercially named Candonga, at two different ripening stages (half-red and red) and at three harvest times (H1, H2 and H3); Figure S1. Half-red (in ripening phase, fully expanded and 50% red), and Red (in ripening phase, fully expanded and 100% red) Candonga strawberries used in the experiment.
