# Supplementary material for: Rapid and Non-Destructive Techniques for the Discrimination of Ripening Stages in Candonga Strawberries
Source: Foods. 2022 May 24;11(11):1534. doi: 10.3390/foods11111534 (PMC9180294; doi:10.3390/foods11111534)
Supplement: Supplementary file 1 [file foods-11-01534-s001.zip › foods-1716936-supplementary.pdf]

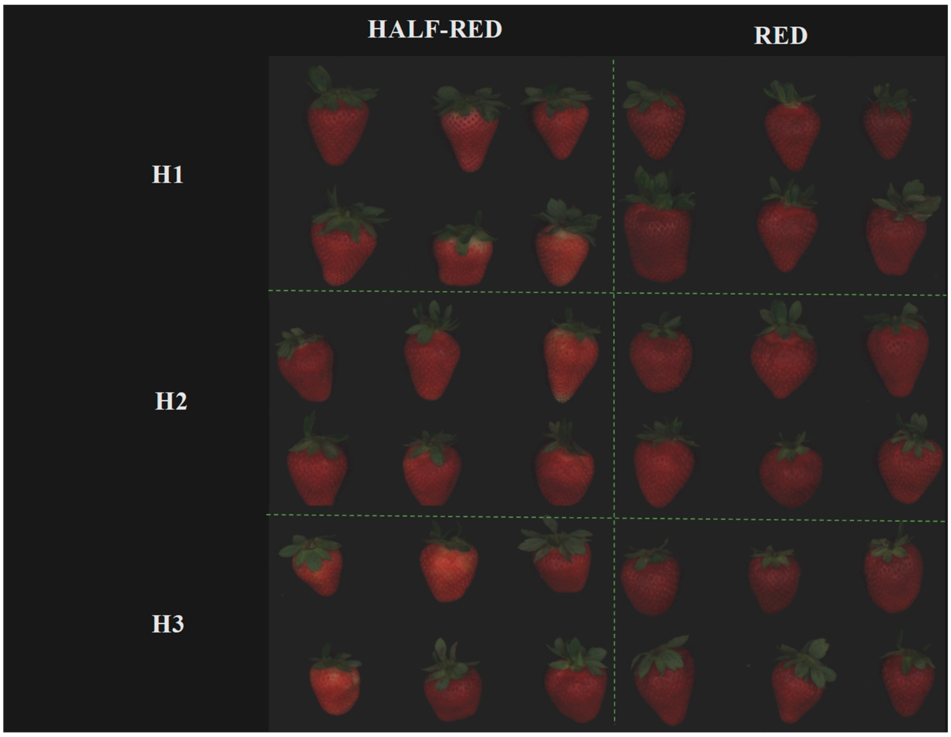

Figure S1. Half-red (in ripening phase, fully expanded and 50% red), and Red (in ripening phase, fully expanded and 100% red) Candonga strawberries used in the experiment.
